# Subitizing endures in sequential rather than simultaneous comparison tasks

**DOI:** 10.1002/pchj.750

**Published:** 2024-04-15

**Authors:** Wei Liu, Chunhui Wang, Jinglin Tian, Guido Marco Cicchini

**Affiliations:** ^1^ College of Education Yunnan Minzu University Kunming China; ^2^ Institute of Neuroscience National Research Council Pisa Italy

**Keywords:** estimation, inverse efficiency score (IES), parallel processing, subitizing, Weber's law

## Abstract

Subitizing is the ability to appraise a number of small quantities (up to four) rapidly and precisely. This system, however, can be impaired by distractors presented along with targets to be enumerated. To better understand whether this limitation arises in perceptual circuits or in the response selection stage, we investigated whether subitizing can endure in simultaneous comparison tasks. Participants were asked to compare the number of dots in two sets on the left and right sides of the screen, presented either simultaneously or sequentially. For comparing within the numerosity range (6–32 dots), both the error rate and reaction time increased steadily as the ratio between the two numbers compared approached “1.” Namely, a phenomenon labeled the ratio effect was revealed. For comparison with small numbers (<5), the sequential comparison task was errorless despite the ratio, suggesting the feature of subitizing. Individual efficiency (measured by the inverse efficiency score [IES]) did not correlate between number ranges in sequential comparison, suggesting that distinct mechanisms were involved. However, we found that in simultaneous tasks, error rate and efficiency showed an increase as the ratios of the two numbers compared approached “1.” This is similar to the ratio effect revealed in the comparison for moderate numbers. Individual efficiency within these two ranges correlated, indicating that the enumeration within these two ranges was based on a single mechanism. These results suggest that subitizing cannot process sets in parallel, and numerosity takes the job whenever subitizing fails.

## INTRODUCTION

The number of items within a moderate number range can be appraised rapidly with a just noticeable difference proportional to the mean of estimation, resulting in a constant Weber fraction and suggesting the activity of the approximate number system (ANS; Anobile et al., 2014, 2016; Dehaene, 2003; Gallistel & Gelman, 2000; Halberda et al., 2006; Ross, 2003). What are the limits of the estimation system? There is apparently a lower limit to estimation as it is reported to work only above five. Below that point there are processes providing fast and perfect performance. Namely, up to four items can be appraised rapidly and precisely by a mechanism dubbed subitizing (Jevons, 1871; Kaufman et al., 1949). Subitizing is widely regarded as one of the most outstanding feats of the human numerosity system, which precisely appreciates very small numerosities. However, there are works showing that in fact estimation is acting even with very few items (Burr et al., 2010; Liu et al., 2020; Pomè, Anobile, Cicchini, Scabia, & Burr, 2019).

According to Weber's law, processing efficiency of estimation depends on the ratio of two numbers being compared, rather than the absolute number value. As the ratio approaches 1, it becomes more and more difficult to judge which set has more items. Reaction time and error rate are found to increase as the ratio goes up (therefore the two numbers to be compared get closer and closer), a phenomenon labeled numerical ratio effect (Bugden & Ansari, 2011; Moyer & Landauer, 1967). However, as soon as numerosity perception was documented, it also became clear that an exception to this rule was that of small numerosities. Performance in the subitizing range is not ratio dependent (Feigenson & Carey, 2003). Subitizing can be distinguished by its relatively flat and low reaction time and constantly near‐to‐zero error rate from the enumeration of moderate numerosities in a counting task (Trick & Pylyshyn, 1993). The performance discontinuity between small and moderate numerosities suggests separate systems for “subitizing” and “estimation” (Anobile et al., 2020; Camos & Tillmann, 2008; Pomè, Anobile, Cicchini, & Burr, 2019; Schleifer & Landerl, 2011). In line with this suggestion, although the abilities of estimation and math are proposed to be highly correlated (Anobile et al., 2013; Cicchini et al., 2016, 2019), the capability of subitizing is revealed to be independent of the abilities of estimation or math (Anobile et al., 2019; but also see the study of Ashkenazi et al., 2013).

The capacity to subitize is highly related to attention (Olivers & Watson, 2008; Palmer et al., 1993). Subitizing vanishes in dual‐task paradigms in which the attention resource for number task is deprived by the primary task or during an attentional blink (Burr et al., 2010, 2011; Pomè, Anobile, Cicchini, Scabia, & Burr, 2019). Interestingly, the system mediating subitizing does not have the capability to process multiple sets of items in parallel. In a previous study, we demonstrated that the simple introduction of a second group of items marked by a different color led to loss of the traits of subitizing (Liu et al., 2020). In that study, 1–5 distinct sets defined by colors were presented simultaneously, and the participants were asked to report the dot number of the target set. They were told which of the color sets was the target either before or after the stimuli display. According to the results, errorless subitizing failed as long as there was more than one set presented. In these very conditions, Weber's law emerged even in the numerosity range 1–4. The typical traits of subitizing were present only when the participants were asked to report the dot number of all dots (superset), or when there was only one color set. All this stands in stark contrast with the performance provided by the approximate number system, which effortlessly processed two simultaneously presented sets as well as their superset even when subjects were post‐cued to the target set (Halberda et al., 2006; Liu et al., 2020). However, it is not clear why subitizing was lost in parallel estimation tasks. In our previous studies, working memory was taxed as well as attention, as subitizing was impaired when subjects had to keep the dot numbers from multiple subsets in mind until the target set was cued. In other literature, the dual task paradigm also taxed working memory when subjects had to remember responses for two tasks (Burr et al., 2010; Pomè, Anobile, Cicchini, Scabia, & Burr, 2019). Therefore, it is unknown whether subitizing fails because of a loading of attentional allocation or a loading in working memory storage.

The current study was aimed at collecting further evidence for the idea that subitizing cannot process sets in parallel, particularly in conditions that tax attention but not working memory, to demonstrate that attentional allocation, rather than working memory load, plays a crucial role in the loss of subitizing. To this end, we employed a simultaneous comparison of numerosity, a paradigm that strains perceptual and attentional resources to capture information in two parts of the visual scene at the same time. In this paradigm, loading of working memory is well controlled, because one can formulate a judgement as soon as one sees the stimuli and hence only one simple binary answer “red” or “green” is proposed to be stored. Inverse efficiency score (IES; i.e., the reaction time divided by accuracy), which combines speed and accuracy into a unified measure to eliminate the speed–accuracy trade‐off, is adopted to assess individual efficiency (Hughes et al., 2014; Vandierendonck, 2017). To this end we found that whilst being distinct in sequential comparison task, error rate and efficiency within subitizing and estimation ranges were identical in the simultaneous comparison task. Furthermore, individual IES within subitizing and numerosity ranges were not correlated in the sequential task, whereas they were correlated in the simultaneous task, suggesting a single mechanism underlying the enumeration within these two regimes. According to these results, parallel activation of the numerosity estimation system is proposed whenever subitizing is not possible. The fact that the estimation system was capable of handling sets with extremely low numerosities suggests that modelling studies that aim at performing estimation should not impose any constraints on the lower limit of the processing capability.

## METHOD

### Sample size and subjects

Subjects aged from 20 to 34 years with normal or corrected‐to‐normal vision and normal color perception were recruited. Sixteen subjects (average age 23.40 years, six males) participated in the experiments. In the subitizing conditions (A, C, and D), the number range of each subset was 1–7, and we focused on the trials in which the dot number of each set was in the range of 1–4, and the two numbers were not equal. The analyzed ratios of two compared numbers (the smaller divided by the bigger) are 0.25, 0.33, 0.5, 0.67, and 0.75. Trials with Target 5–7 were eliminated from the analysis because these numbers are beyond the subitizing range. A total of 11,760 trials were run, and 2880 trials were analyzed in each subitizing condition. This apparently inefficient paradigm was chosen so to avoid the ubiquitous range effects (Cicchini et al., 2022). If we never showed numerosities beyond four, observers would easily infer the tested range and would have a strong cue not to overestimate number four (as this would have been the highest number of the range).

In numerosity conditions (B and E), the number range of each subset was 4–42, and trials within 6–32 with the previous number ratios were analyzed. The biggest numbers were eliminated from the analysis to make the comparison analogous to that for small numbers. Moreover, trials with number ratios other than 0.25, 0.33, 0.50, 0.67, and 0.75 were removed, so that a direct comparison could be made between the two ranges. A total of 3795 trials were run and 2250 trials were analyzed in each numerosity condition. These experimental parameters were chosen following previous studies (Liu et al., 2020) that indicated reliable *p*‐values and Bayes factors with those numbers of trials and subjects. Based on a power analysis (using software G*Power; Faul et al., 2007), the minimal required sample size to decide the effect of interaction is 11 participants (analysis of variance [ANOVA], repeated‐measures, within factors, six measurements, alpha = .05, power = 0.80). Hence sufficient participants (*N* = 16) were recruited in the current study.

### Stimuli and procedure

Subjects sat approximately 50 cm from an LCD monitor with a viewable area measuring 41 cm by 26 cm (19″, 1920 × 1080, 60 Hz) in a dimly lit quiet room. Stimuli were generated from Matlab (MathWorks). On each trial, the dots could be classified into two sets by their colors (red or green). The sets with different colors also had different shapes (square or circle), but the shape was not a relevant attribute to the tasks. The diameter of each square was 0.5° visual angle (20 pixels), and the diameter of each circle was 0.55° (22 pixels). Dots were presented in a circle at the center of the screen with a diameter of 13.6° (542 pixels) visual angle in A, C, and D, and in a much larger circle with a diameter of 23.2 ° (930 pixels) visual angle in B and E, because far more dots were displayed in these two conditions.

**FIGURE 1:**
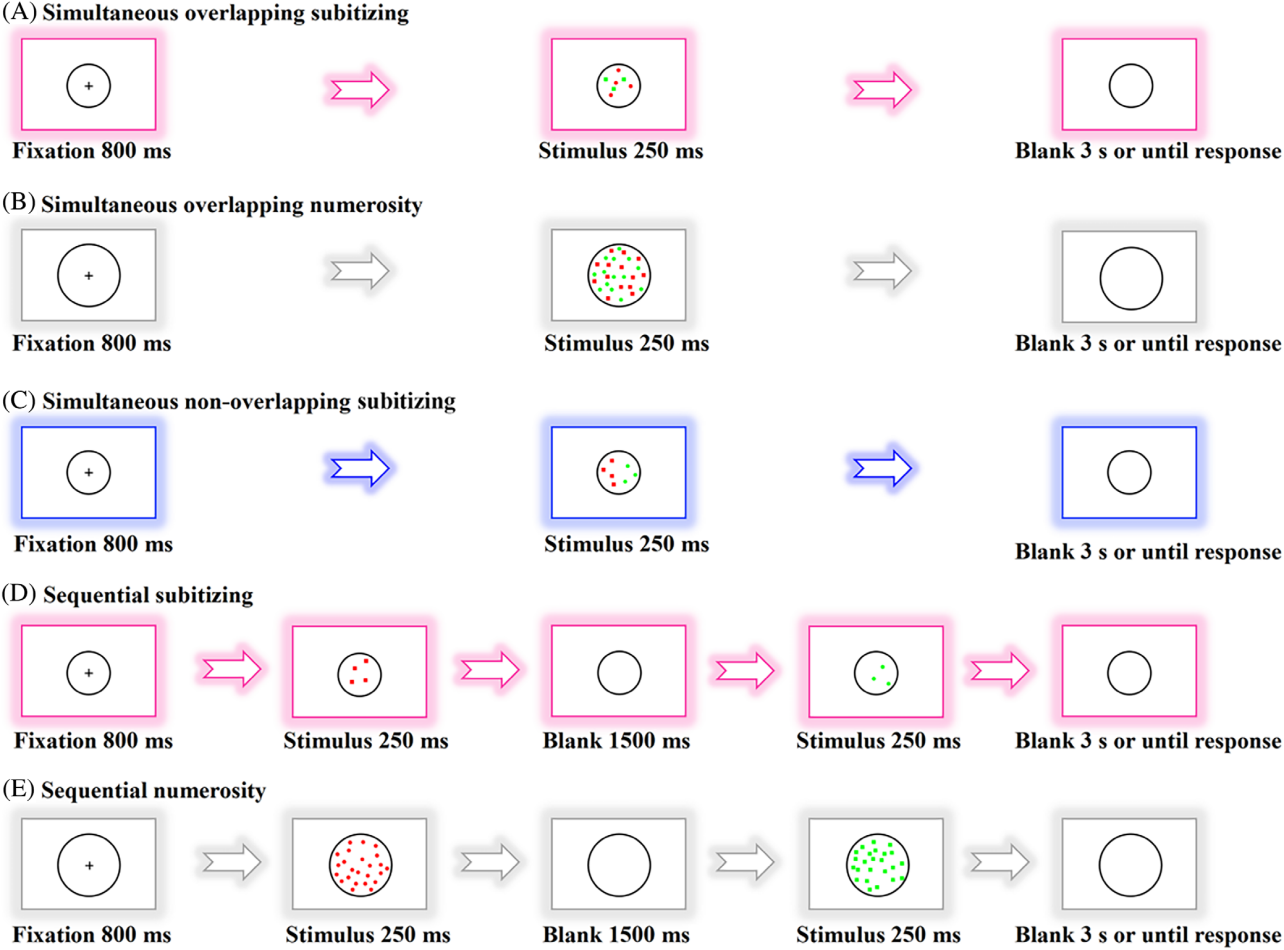
Schematic illustration describing the conditions of simultaneous and sequential comparisons. Parts (A–C) illustrate the procedure of simultaneous presentation within the (A, C) subitizing (1–4) and (B) numerosity (6–32) regimes. Subjects were asked to state which colored group had the higher numerosity. Dots were spatially overlapping in Conditions A and B, whereas the two color subsets were spatially separated in Condition C. Parts (D, E) demonstrate the procedure of sequential presenting condition in the two number regimes.

Figure 1 shows the experimental procedure in each condition. On each trial of simultaneous conditions (A–C), subjects saw a fixation lasting for 800 ms, followed by a 250‐ms display containing red and green dots. They compared the number of two‐color subsets and chose which colored set contained more dots as quickly as possible (2AFC paradigm). In sequential conditions (D and E), the first stimulus containing dots of a single color in each display (red or green) lasted for 250 ms, followed by a second display that showed the other color (green or red) and lasted for 250 ms. The two stimulus displays were separated by a blank lasting for 1500 ms. Subjects were asked to choose which color subset was more numerous after they saw the second display. The order of the test conditions was counterbalanced across participants.

Dots in the stimulus patch were randomly distributed in the circle and they did not overlap with each other. In Condition A and B, the dots of two colors were spatially overlapping. In Condition C, however, they were spatially separated. Note that we further examined the non‐overlapping subitizing condition to figure out whether subitizing re‐emerges when there is no spatial overlapping, even in the simultaneous comparison task. Hence a non‐overlapping comparison was not investigated within the numerosity range. The number of dots in each color subset was randomly determined within its number range with the constraint that the trials for each number pair were equal. In subitizing conditions, there were from 30 to 60 trials analyzed for each ratio and each subject. In numerosity conditions, there were from 20 to 60 trials analyzed.

### Data analysis

Error rate (ER; wrong trials divided by total trials), reaction time for correct response trials (RT), and efficiency or the IES (i.e., the average correct RT divided by the accuracy rate) were calculated for each subject under each condition. Both in the simultaneous and sequential presentation conditions, repeated‐measures ANOVA was conducted with number regime (subitizing/numerosity) and number ratio (0.5/0.67/0.75) as independent variables. Respectively, ER, RT, and IES were analyzed as dependent variables of ANOVA. $\eta_{p}^{2}$ was reported to estimate the effect of independent variables. Paired‐tests of Pearson correlation for individual IES were conducted between each two tasks. Bayes factors (*BF*_10_) were reported to estimate whether the null hypothesis H_0_ or the alternative hypothesis H_1_ was more likely to be correct. *BF*_10_ < 0.3 suggested clear evidence for H_0_, whereas *BF*_10_ > 3 indicated clear evidence for H_1_. The false discovery rate probability (FDR), or *Q*‐value, was adopted in this study to correct the likelihood of type‐I error in multiple comparisons. For example, with four *p*‐values in multiple comparisons, we multiplied the smallest *p*‐value by four to get its *Q*‐value. Then we multiplied the second smallest *p* by 4/2, the third *p* by 4/3, and the last *p* (the largest one) by 4/4 to get their *Q*‐values. To determine whether a comparison was statistically significant, *Q*‐values were used instead of *p*‐values. *Q* = 0.05 is a widely accepted threshold for significance. JASP and SPSS 16.0 were used for statistics. Data from all participants were included.

## RESULTS

### Simultaneous comparisons

**FIGURE 2:**
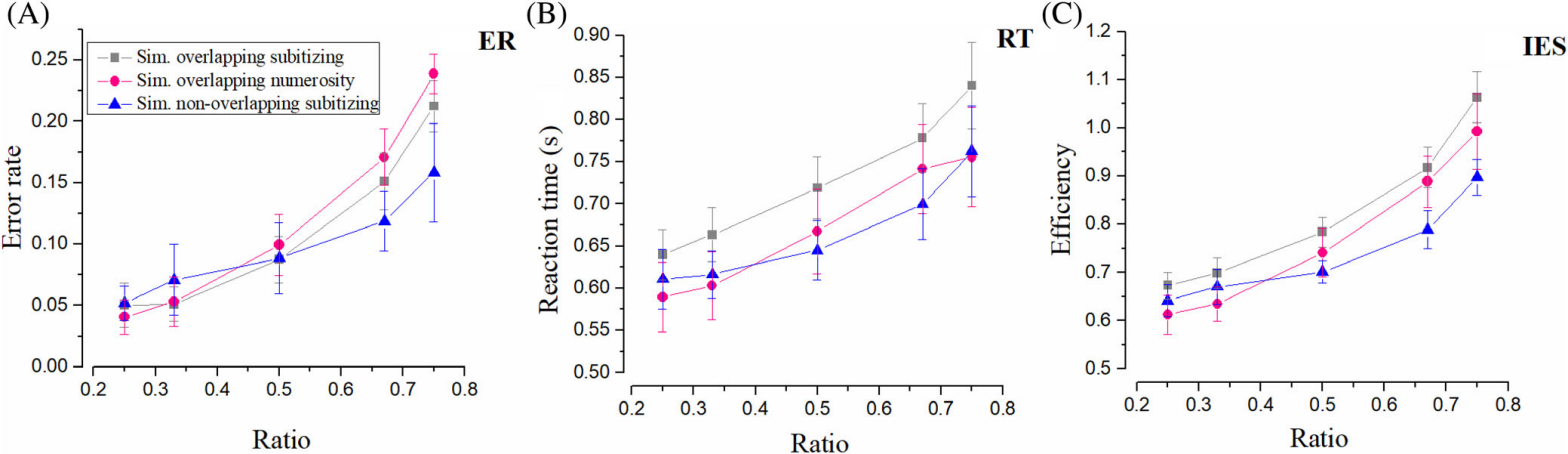
Results in simultaneous conditions. “Sim.” is the shortened form of “Simultaneous.” (A) Error rate (ER) as function of numerical ratio of the two sets. Gray symbols represent the ER of simultaneous overlapping comparison within the subitizing range 1–4. Pink symbols demonstrate the results of ER in simultaneous comparison within the numerosity range of 6–32. Blue represents the ER in a simultaneous non‐overlapping comparison. (B) Same for reaction time (RT). (C) Results of efficiency (inverse efficiency score [IES]). Please note that genuine subitizing implies constant (and low) error rates and reaction times.

Figure 2 displays ER, RT, and IES in simultaneous conditions. Gray lines stand for the subitizing range in Condition A, pink ones stand for the numerosity range in Condition B, and blue ones show the results of non‐overlapping subitizing in Condition C.

#### Simultaneous overlapping sets: Subitizing versus estimation ranges (A vs. B)

We first compared judgements of overlapping sets in the subitizing and estimation range (Experiment A and B). We show three dependent measures (Error Rate, RT, and Efficiency), respectively, in Figure 2A–C. Repeated‐measures ANOVA with ER as the dependent variable returned no significant main effect of number range (subitizing/numerosity), *F*(1, 15) = 1.49, *p* = .241, $\eta_{p}^{2}$ = .09, *BF*_10_ = 0.56, a significant main effect of ratio (0.5/0.67/0.75), *F*(2, 30) = 35.26, p < .001, $\eta_{p}^{2}$ = .70, *BF*_10_ > 100, and no significant interaction, *F*(2, 30) = 0.12, *p* = .887, $\eta_{p}^{2}$ = .01, *BF*_10_ = 0.19. When reaction time was taken as the dependent variable, repeated‐measures ANOVA revealed a significant main effect of number range, *F*(1, 15) = 5.34, *p* = .035, $\eta_{p}^{2}$ = .26, *BF*_10_ > 100, a significant main effect of ratio, *F*(2, 30) = 24.92, *p* < .001, $\eta_{p}^{2}$ = .62, *BF*_10_ > 100, and a significant interaction, *F*(2, 30) = 5.00, *p* = .013, $\eta_{p}^{2}$ = .25, *BF*_10_ = 0.32. Note that no evidence for interaction was suggested by Bayes factors.

Error rate and reaction time are two important indicators to quantify the performance. However, potential contradictory effects may exist in these two dimensions because of the speed–accuracy trade‐off. The IES, a unified measure combing RT and ER together, is suggested to be more reliable in reflecting the efficiency of judgement (Kerzel, 2019; Liu et al., 2019; Vandierendonck, 2017). When IES was taken as the dependent variable, repeated‐measures ANOVA revealed no significant main effect of number range, *F*(1, 15) = 1.58, *p* = .229, $\eta_{p}^{2}$ = .10, *BF*_10_ = 1.07, a significant main effect of ratio, *F*(2, 30) = 43.46, *p* < .001, $\eta_{p}^{2}$ = .74, *BF*_10_ > 100, and no significant interaction, *F*(2, 30) = 0.51, *p* = .605, $\eta_{p}^{2}$ = .03, *BF*_10_ = 0.18.

In Condition B, the comparison was supposed to be based on numerosity. According to Weber's law, the processing efficiency of estimation depends on the ratio of two numbers being compared. IES increases as the ratio increases, revealing the numerical ratio effect. This effect is expected to be greatly reduced if comparisons between small numbers are based on subitizing, since performance in subitizing ranges is not ratio‐dependent. However, no interaction was found between Condition A and B, suggesting an identical numerical ratio effect or processing efficiency, regardless of the number range. In other words, simultaneous comparison between small numbers is suggested to be governed by Weber's law similar to moderate numbers.

**FIGURE 3:**
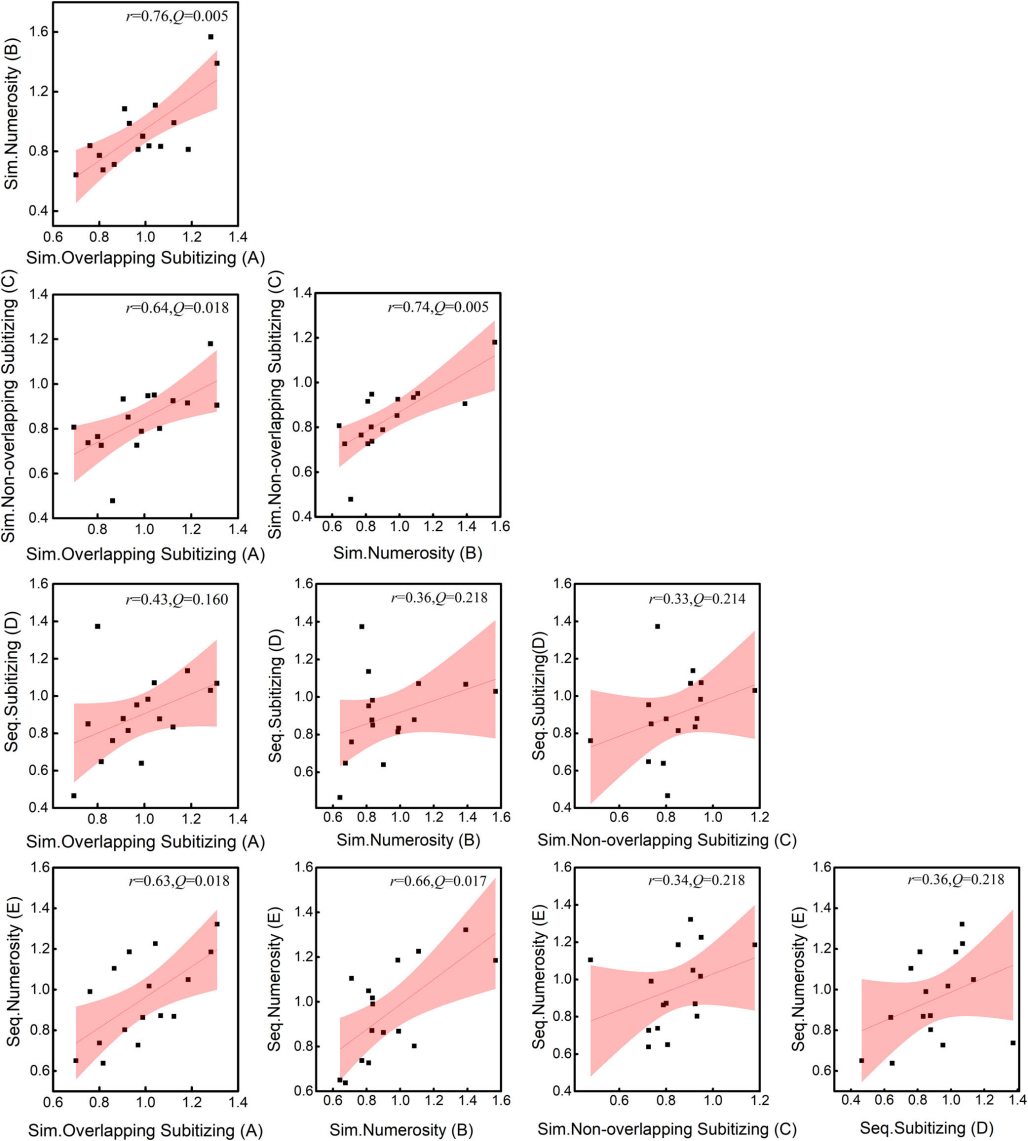
Scatters for individual inverse efficiency score (IES) of the two highest ratios (0.67 and 0.75) between each two conditions. The *Q*‐value represents the corrected probability of type‐I error in multiple comparisons. Pink area denotes 95% confidence interval.

To better highlight the tight link between the performance in the two regimes, we isolated efficiencies or IES in the most difficult comparisons, those with the highest ratios (0.67 and 0.75), and compared them across the two regimes (Table 1, Figure 3). This analysis revealed a significant correlation between subitizing and numerosity tasks, *r* = 0.76, *p <* .001, *BF*_10_ = 54.47, *Q* = 0.005. It is suggested by the results that despite the number range, a single mechanism is responsible for the simultaneous comparison.

**TABLE 1:** Correlation of individual efficiencies (IES) in task pairs of the current dataset.

Conditions	Sim. Overlapping subitizing (A)	Sim. Numerosity (B)	Sim. Non‐overlapping subitizing (C)	Seq. Subitizing (D)
Sim. Numerosity (B)	*r* = 0.76, *p* < .001 *BF*_10_ = 54.47, *Q* = 0.005**			
Sim. Non‐overlapping Subitizing (C)	*r* = 0.64, *p* = .008	*r* = 0.74, *p* = .001		
	*BF*_10_ = 7.81, *Q* = 0.018*	*BF*_10_ = 43.62, *Q* = 0.005**		
Seq. Subitizing (D)	*r* = 0.43, *p* = .096	*r* = 0.36, *p* = .174	*r* = 0.33, *p* = .214	
	*BF*_10_ = 1.11, *Q* = 0.160	*BF*_10_ = 0.73, *Q* = 0.218	*BF*_10_ = 0.63, *Q* = 0.214	
Seq. Numerosity (E)	*r* = 0.63, *p* = .009	*r* = 0.66, *p* = .005	*r* = 0.34, *p* = .196	*r* = 0.36, *p* = .168
	*BF*_10_ = 7.06, *Q* = 0.018*	*BF*_10_ = 10.77, *Q* = 0.017*	*BF*_10_ = 0.67, *Q* = 0.218	*BF*_10_ = 0.74, *Q* = 0.218

*Note*: The average IESs of the two highest ratios (0.67 and 0.75) are compared across the two regimes. We only reported IES correlation because it provides a more reliable assessment of individual efficiency by combining speed and accuracy into a unified measure, which eliminates the speed–accuracy trade‐off. *Q*‐value stands for the false discovery rate probability (FDR), or the corrected probability of type‐I error in multiple comparisons. “Sim.” is the shortened form of “Simultaneous” and “Seq.” is the shortened form of “Sequential.”

* *Q* < 0.05;

** *Q* < 0.01.

#### Simultaneous overlapping versus non‐overlapping sets: Subitizing range (A vs. C)

We next compared Experiment A and Experiment C to investigate whether subitizing re‐emerges in non‐overlapping presentations and if the two performances correlate with each other. As for ER, repeated‐measures ANOVA revealed no significant main effect of display (overlap/non‐overlap), *F*(1, 15) = 1.65, *p* = .219, $\eta_{p}^{2}$ = .10, *BF*_10_ = 1.14, a significant main effect of ratio (0.5/0.67/0.75), *F*(2, 30) = 29.92, *p <* .001, $\eta_{p}^{2}$ = .67, *BF*_10_ > 100, and no significant interaction, *F*(2, 30) = 1.59, *p* = .220, $\eta_{p}^{2}$ = .10, *BF*_10_ = 0.39. Repeated‐measures ANOVA for reaction time revealed a significant main effect of display, *F*(1, 15) = 6.53, *p* = .022, $\eta_{p}^{2}$ = .30, *BF*_10_ > 100, a significant main effect of ratio, *F*(2, 30) = 26.04, *p* < .001, $\eta_{p}^{2}$ = .63, *BF*_10_ > 100, and no significant interaction, *F*(2, 30) = 0.05, *p* = .955, $\eta_{p}^{2}$ = .003, *BF*_10_ = 0.16.

As for IES, repeated‐measures ANOVA revealed a significant main effect of display, *F*(1, 15) = 15.18, *p* = .001, $\eta_{p}^{2}$ = .50, *BF*_10_ > 100, a significant main effect of ratio, *F*(2, 30) = 74.83, *p* < .001, $\eta_{p}^{2}$ = .83, *BF*_10_ > 100, and no significant interaction, *F*(2, 30) = 3.12, *p* = .059, $\eta_{p}^{2}$ = .17, *BF*_10_ = 0.45. Paired‐test for individual efficiency of highest ratios (0.67 and 0.75) revealed a significant correlation between overlapping and non‐overlapping conditions, *r* = 0.64, *p* = .008, *BF*_10_ = 7.81, *Q* = 0.018.

Condition A and C are distinct in their presentation of stimuli. In spite of this, no IES interaction was detected in ANOVA analyses, and the IES was significantly correlated, indicating identical processing efficiency and a single mechanism for these simultaneous tasks.

#### Simultaneous overlapping estimation versus non‐overlapping subitizing ranges (B vs. C)

We made further effort to investigate simultaneous comparison within the subitizing range under a condition in which two color subsets were confined to two sides of one circle and no longer overlapped (Condition C). The results are compared with Condition B to test whether simultaneous comparison IES in subitizing and numerosity range could be correlated, even when two color sets were presented differently in spatial overlap. ER, RT, and IES in these two conditions can be found in Figure 2 (pink and blue symbols). Under such conditions, as for ER, repeated‐measures ANOVA revealed a significant main effect of number range (subitizing/numerosity), *F*(1, 15) = 7.59, *p* = .015, $\eta_{p}^{2}$ = .34, *BF*_10_ = 34.36, a significant main effect of ratio (0.5/0.67/0.75), *F*(2, 30) = 40.36, *p <* .001, $\eta_{p}^{2}$ = .73, *BF*_10_ > 100, and no significant interaction, *F*(2, 30) = 1.89, *p* = .169, $\eta_{p}^{2}$ = .11, *BF*_10_ = 0.71. Repeated‐measures ANOVA for reaction time revealed no significant main effect of number range, *F*(1, 15) = 0.38, *p* = .546, $\eta_{p}^{2}$ = .03, *BF*_10_ = 0.37, a significant main effect of ratio, *F*(2, 30) = 21.45, *p* < .001, $\eta_{p}^{2}$ = .59, *BF*_10_ > 100, and a significant interaction, *F*(2, 30) = 5.78, *p* = .008, $\eta_{p}^{2}$ = .28, *BF*_10_ = 0.26.

As for IES, repeated‐measures ANOVA revealed no significant main effect of number range, *F*(1, 15) = 3.64, *p* = .076, $\eta_{p}^{2}$ = .20, *BF*_10_ = 15.20, a significant main effect of ratio, *F*(2, 30) = 46.74, *p* < .001, $\eta_{p}^{2}$ = .76, *BF*_10_ > 100, and no significant interaction, *F*(2, 30) = 1.21, *p* = .314, $\eta_{p}^{2}$ = .07, *BF*_10_ = 0.23. Paired‐test for individual efficiency of highest ratios (0.67 and 0.75) revealed a significant correlation between subitizing and numerosity, *r* = 0.74, *p* = .001, *BF*_10_ = 43.62, *Q* = 0.005. Similar to previous analyses, both the lack of interaction and the significant correlation suggest that processing in these two tasks is tightly linked, even when the two color sets were presented in different spatial overlap conditions, and the dots in these two tasks were within different number ranges.

### Sequential comparisons

#### Subitizing versus estimation ranges (D vs. E)

**FIGURE 4:**
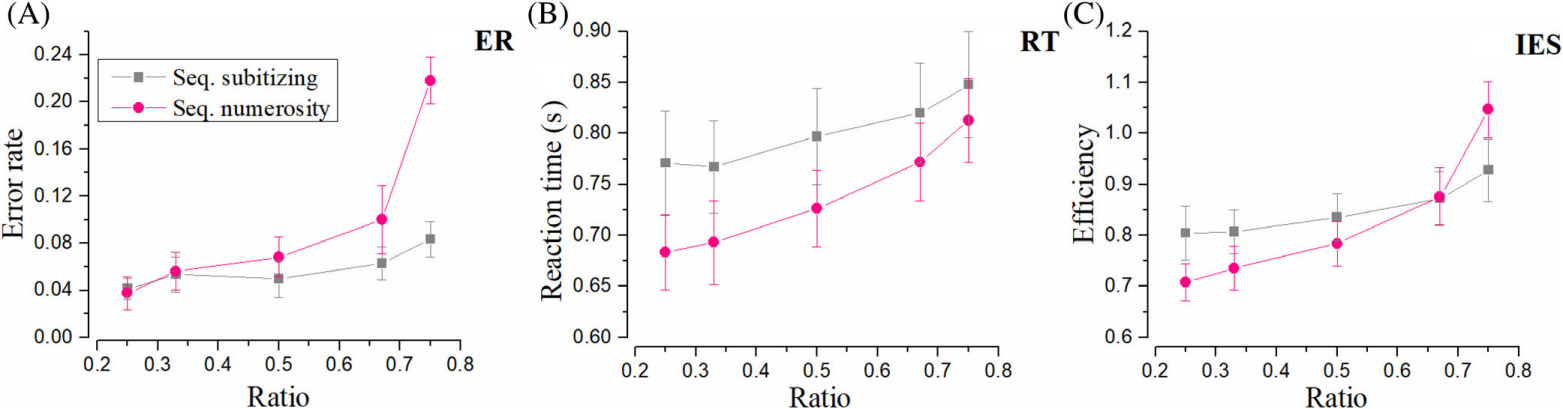
Results in sequential conditions. “Seq.” is the shortened form of “Sequential.” (A) Results of error rate (ER). Gray symbols illuminate the ER of sequential comparison within the subitizing range 1–4. Pink symbols demonstrate the results of ER sequential comparison in the numerosity range 6–32. (B) Results of reaction time (RT). (C) Results of efficiency (inverse efficiency score [IES]).

In most previous studies, subitizing could only be revealed in the estimation task. However, in this study, the processing dissociation between small (<5) and moderate number ranges was revealed in sequential tasks, suggesting that the mechanism underlying sequential comparison is highly related to subitizing. Figure 4 shows ER, RT, and IES in sequential conditions. Error rates in this condition in the subitizing range (pink) are much flatter. Indeed, repeated‐measures ANOVA revealed a significant main effect of number range, *F*(1, 15) = 9.59, *p* = .007, $\eta_{p}^{2}$ = .39, *BF*_10_ > 100, and a significant main effect of ratio, *F*(2, 30) = 24.00, *p <* .001, $\eta_{p}^{2}$ = .62, *BF*_10_ > 100. A significant interaction was found, *F*(2, 30) = 9.58, *p <* .001, $\eta_{p}^{2}$ = 0.39, *BF*_10_ = 22.70, indicating that the two number ranges operate differently when number ratio varies. Repeated‐measures ANOVA for reaction time revealed a significant main effect of number range, *F*(1, 15) = 1.64, *p* = .022, $\eta_{p}^{2}$ = .10, *BF*_10_ = 3.94, a significant main effect of ratio, *F*(2, 30) = 17.06, *p* < .001, $\eta_{p}^{2}$ = .53, *BF*_10_ = 2.24, and no significant interaction, *F*(2, 30) = 1.19, *p* = .318, $\eta_{p}^{2}$ = .07, *BF*_10_ = 0.19. Noticeably, sequential comparison within subitizing range was significantly slower, rather than faster than that within estimation range.

As for IES, repeated‐measures ANOVA revealed no significant main effect of number range, *F*(1, 15) = 0.20, *p* = .659, $\eta_{p}^{2}$ = .01, *BF*_10_ = 0.28, and a significant main effect of ratio, *F*(2, 30) = 39.89, *p* < .001, $\eta_{p}^{2}$ = .73, *BF*_10_ > 100. Importantly, a significant interaction was found, *F*(2, 30) = 7.66, *p =* .002, $\eta_{p}^{2}$ = .34, *BF*_10_ = 1.56. Paired‐test for individual efficiency with higher ratios revealed no significant correlation between subitizing and numerosity tasks, *r* = 0.36, *p* = .168, *BF*_10_ = 0.74, *Q* = 0.218.

Crucially, for Condition D and E, all experiment parameters were identical in all aspects except the number range. However, ANOVA analyses revealed a significant interaction in IES. It is suggested that processing efficiency varies. IES for moderate numbers followed Weber's law, which increased rapidly with increasing ratios. For small numbers, however, the IES remained relatively flat regardless of the ratio, and was not correlated with that for moderate numbers. These results provide clear evidence that distinct mechanisms are involved in sequential comparison within small and moderate number ranges.

## CONTROL EXPERIMENT

There is a concern that estimation for very small numerosities could be based on magnitude rather than number (e.g., subjects make a decision by comparing the cumulative area of dots). In the main experiment, the dots in the two sets were different in shape, and the circles were apparently smaller than the squares, although their physical area was matched, probably because the perceived area could be decided by the additive area rather than the mathematical area (Yousif & Keil, 2020). However, there was no trend suggesting that larger squares seemed to be more numerous.

**FIGURE 5:**
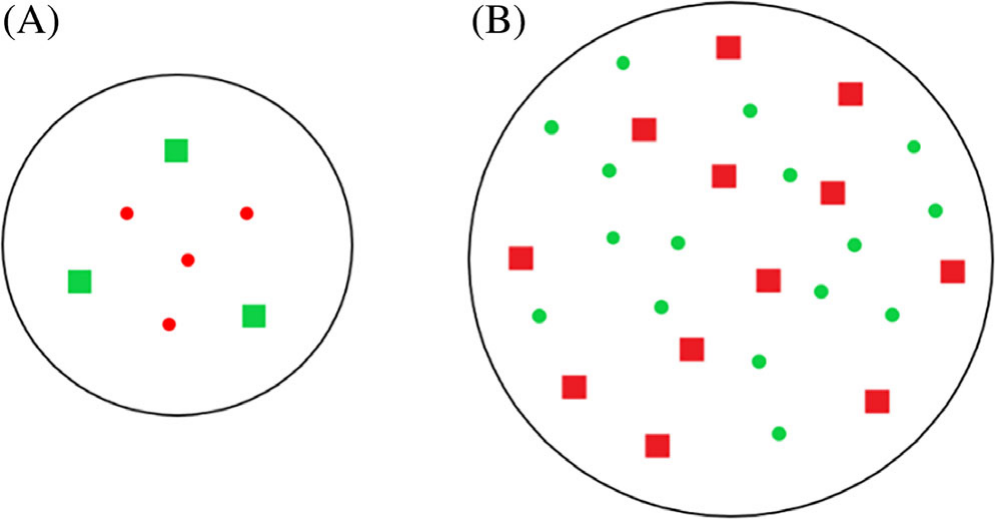
Stimuli in the control experiment. Each circle was decreased to 56% of its area compared to that used in the main experiment, whereas the square size was kept constant. Hence three circles were smaller than two squares (0.67), and four circles were smaller than three squares (0.75) in the accumulative area. With these ratios, the cue of area and the cue of number were inconsistent in half of the trials. Figure 5A illustrates the stimuli within subitizing regimes, and Figure 5B shows the stimuli within numerosity regimes.

To further exclude the possibility that the interaction between simultaneous/sequential comparison and small/moderate numbers was due to magnitude, such as area rather than number processing, we asked 12 participants (average age 25 years, four males) to take part in a control experiment in which the circle size was further reduced by 44%, while the square size was kept constant (Figure 5). Hence three circles were smaller than two squares (0.67), and four circles were smaller than three squares (0.75) in the accumulative area. With these ratios, the cue of area and the cue of number were inconsistent in half of the trials. Participants were asked to conduct the simultaneous subitizing/numerosity comparison and the sequential subitizing/numerosity comparison tasks in a counterbalanced order. The trial number and data analyses were identical to the main experiment.

**FIGURE 6:**
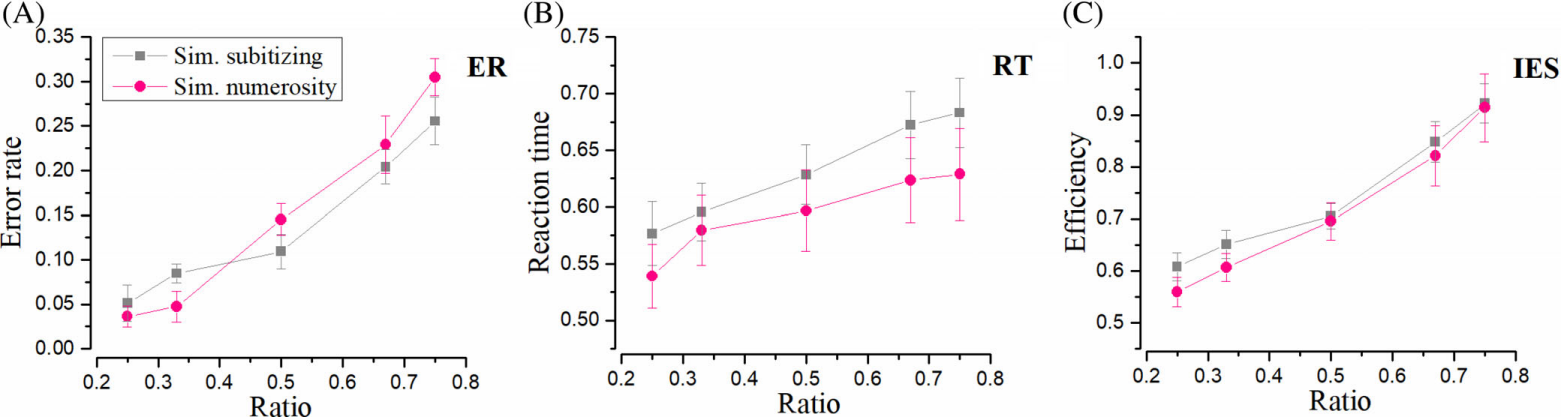
Results in simultaneous conditions in the control experiment. “Sim.” is the shortened form of “Simultaneous.” (A) Error rate (ER) as a function of the numerical ratio of the two sets. Gray symbols represent the ER of simultaneous overlapping comparison within the subitizing range of 1–4. Pink symbols demonstrate the results of ER in simultaneous comparison within the numerosity range of 6–32. (B) Results of reaction time (RT). (C) Results of efficiency (inverse efficiency score [IES]).

Figure 6 shows the results of simultaneous comparison. Note that in the control experiment, the error rate should be 50% for the ratios of 0.67 and 0.75 if the subjects made decisions on magnitude (area) rather than number without noise, because on half of these trails, the circles were smaller than the squares in accumulative area, even if they were more numerous. However, the error rate still stayed around 25% with these ratios, both for subitizing and numerosity conditions. Compared to the formal experiment, the ER in the simultaneous comparison was slightly higher, indicating the influence of the area–number inconsistency. Even with the conflicting visual cues, the RT and IES were even lower than those of the formal experiment, suggesting that processing efficiency remained unaffected.

Importantly, similar ER, RT, and IES interaction was found in the control condition, even when the cue of area was invalid in the comparison tasks. Repeated‐measures ANOVA with ER as a dependent variable yielded no significant main effect of number range (subitizing/numerosity), *F*(1, 11) = 2.35, *p* = .154, $\eta_{p}^{2}$ = .18, *BF*_10_ = 1.85, a significant main effect of ratio (0.5/0.67/0.75), *F*(2, 22) = 26.02, *p* < .001, $\eta_{p}^{2}$ = .70, *BF*_10_ > 100, and no significant interaction, *F*(2, 22) = 0.30, *p* = .747, $\eta_{p}^{2}$ = .03, *BF*_10_ = 0.20. When RT was taken as a dependent variable, repeated‐measures ANOVA revealed no significant main effect of number range, *F*(1, 11) = 2.41, *p* = .149, $\eta_{p}^{2}$ = .18, *BF*_10_ = 1.12, a significant main effect of ratio, *F*(2, 22) = 16.56, *p* < .001, $\eta_{p}^{2}$ = .60, *BF*_10_ = 17.50, and no significant interaction, *F*(2, 22) = 0.95, *p* = .403, $\eta_{p}^{2}$ = .08, *BF*_10_ = 0.22.

When IES was taken as a dependent variable, repeated‐measures ANOVA revealed no significant main effect of number range, *F*(1, 11) = 0.20, *p* = .662, $\eta_{p}^{2}$ = .02, *BF*_10_ = 0.30, a significant main effect of ratio, *F*(2, 22) = 28.41, *p* < .001, $\eta_{p}^{2}$ = .72, *BF*_10_ > 100, and no significant interaction, *F*(2, 22) = 0.08, *p* = .927, $\eta_{p}^{2}$ = .01, *BF*_10_ = 0.23.

**FIGURE 7:**
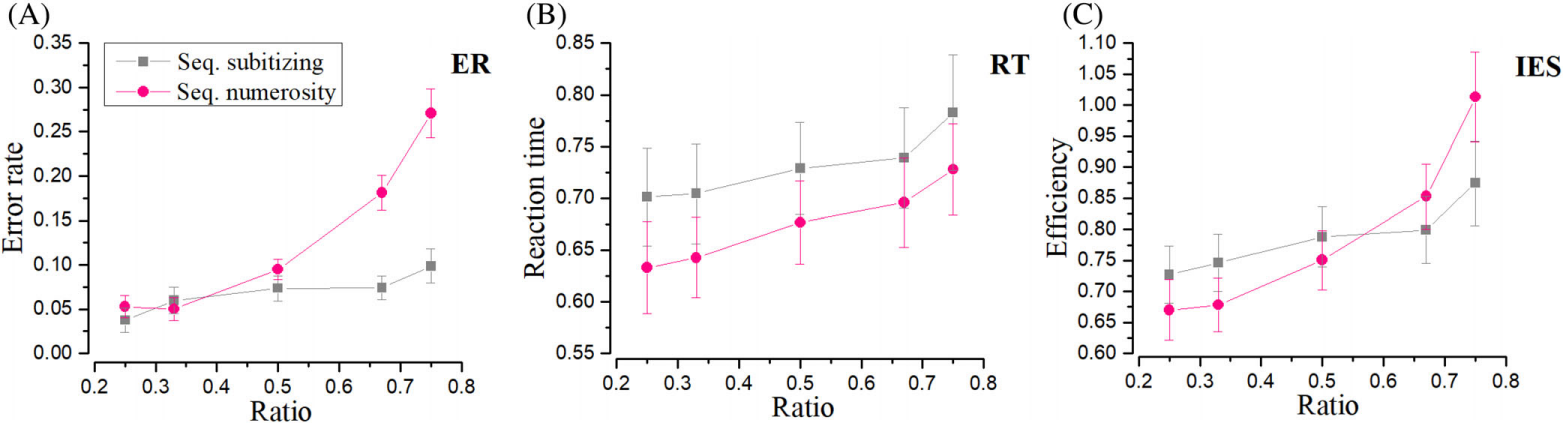
Results in sequential conditions in the control experiment. “Seq.” is the shortened form of “Sequential.” (A) Results of error rate (ER). Gray symbols illuminate the ER of sequential comparison within the subitizing range 1–4. Pink symbols demonstrate the results of ER sequential comparison in the numerosity range 6–32. (B) Results of reaction time (RT). (C) Results of efficiency (inverse efficiency score [IES]).

Figure 7 indicates the results of sequential comparison. The error rates in this condition in the subitizing range (pink) are flat. Indeed repeated‐measures ANOVA revealed a significant main effect of number range, *F*(1, 11) = 50.66, *p* < .001, $\eta_{p}^{2}$ = .82, *BF*_10_ > 100, and a significant main effect of ratio, *F*(2, 22) = 15.87, *p <* .001, $\eta_{p}^{2}$ = .59, *BF*_10_ > 100. A significant interaction was also found, *F*(2, 22) = 15.12, *p <* .001, $\eta_{p}^{2}$ = .58, *BF*_10_ > 100, indicating that the two number ranges operate differently when the number ratio varies. Repeated‐measures ANOVA for reaction time revealed a significant main effect of number range, *F*(1, 11) = 6.35, *p* = .028, $\eta_{p}^{2}$ = .37, *BF*_10_ = 80.55, a significant main effect of ratio, *F*(2, 22) = 8.75, *p* = .002, $\eta_{p}^{2}$ = .44, *BF*_10_ = 7.90, and no significant interaction, *F*(2, 22) = 0.10, *p* = .905, $\eta_{p}^{2}$ = .01, *BF*_10_ = 0.19.

As for IES, repeated‐measures ANOVA revealed no significant main effect of number range, *F*(1, 11) = 2.58, *p* = .137, $\eta_{p}^{2}$ = .19, *BF*_10_ = 1.71, and a significant main effect of ratio, *F*(2, 22) = 22.99, *p* < .001, $\eta_{p}^{2}$ = .68, *BF*_10_ > 100. Importantly, a significant interaction was also found, *F*(2, 22) = 10.83, *p <* .001, $\eta_{p}^{2}$ = .50, *BF*_10_ = 6.57.

In summary, similar ER, RT, and IES interaction was again found in the control experiment, even when the cue of area was invalid in the comparison tasks. In other words, there is no evidence supporting that the interaction between simultaneous/sequential comparison and small/moderate numbers, which was revealed in the formal experiment, is due to the processing of magnitudes such as accumulative area.

## DISCUSSION

We investigated in a series of experiments whether the presentation of a distracting set had a deleterious effect on numerical perception of very small sets even in a simultaneous comparison. Previous evidence from the same authors indicated that when subjects have to enumerate one of two sets consisting of very few items, the typical hallmarks of subitizing are lost if subjects are told which set to judge only after stimulus presentation (Liu et al., 2020). That work opened the door for multiple interpretations. In particular, it was not clear whether the loss of the subitizing was due to an augmented load in working memory or in stimulus processing. In this experiment, we reported very similar results, albeit employing a simultaneous comparison paradigm, which taxes the working memory system much less. This clearly rules out the interpretation that assumes working memory limitations and circumscribes the critical factor to the attentional domain.

Weber's law suggests a processing efficiency depending on the ratio, rather than the absolute value, of two numbers being compared. Both the sequential and simultaneous comparison tasks within numerosity range show a rapid increase of ER and RT as the ratio increases, in line with the proposal in the ratio effect (Bugden & Ansari, 2011; Moyer & Landauer, 1967). On the other hand, given the flat RT and constantly near‐to‐zero just noticeable difference or ER as number increases from one to four in a counting task (Trick & Pylyshyn, 1993), one should infer that the difficulty of comparing “one” with “two,” “two” with “three,” or “three” with “four” does not increase as fast as the ratio grows from 0.5 to 0.75, which is compatible with the slower increases in ER, RT, and consequently IES, as ratio increases, as was found in the sequential subitizing comparison in the current study (Condition D). Individual data analysis suggests that individual IES in sequential tasks within subitizing and numerosity ranges (Condition D vs. E) are not correlated with each other, and that sequential subitizing IES does not correlate with simultaneous numerosity IES (Condition D vs. B), either, which are in line with the previous study stating that the capability of subitizing is not correlated with that of numerosity (Anobile et al., 2019).

Subitizing, whilst being fast and error free, is proposed to be fragile (Burr et al., 2010, 2011; Pomè, Anobile, Cicchini, Scabia, & Burr, 2019). It does not have the capability to process sets in parallel and can be hampered by the introduction of competing sets (Liu et al., 2020). The current study shows further evidence to support these proposals. Noticeably, individual IES within subitizing range are not correlated between simultaneous and sequential tasks (Condition A vs. D; C vs. D). Within the subitizing range, repeated‐measures ANOVA for IES revealed a significant interaction between tasks (simultaneous/sequential) and ratios (0.5/0.67/0.75), *F*(2, 30) = 13.02, *p* < .001, $\eta_{p}^{2}$ = .47, *BF*_10_ = 3.07. These results suggest that subitizing is impaired. Its efficiency, especially for the highest ratios, is affected by the simultaneous comparison task. On the contrary, the processing efficiency of numerosity seems to be unaffected by comparison tasks. When IES is compared between simultaneous and sequential tasks (B vs. E), repeated ANOVA shows no significant main effect for tasks, *F*(1, 15) = 0.35, *p* = .562, $\eta_{p}^{2}$ = .02, *BF*_10_ = 0.32, and no significant interaction, either, *F*(2, 30) = 1.08, *p* = .351, $\eta_{p}^{2}$ = .07, *BF*_10_ = 0.23.

What happens when subitizing fails? One possibility is that in the more taxing conditions perception still relies on the same mechanisms that enable subitizing, albeit with higher noise levels. An alternative possibility is that in the more taxing conditions, a different perceptual system is recruited. We hold that the current dataset supports the latter scenario for two reasons. The first point is the fact that in simultaneous tasks, both ER and IES show an identical pattern, with nearly linear growth as the ratio of two numbers increases. This contrasts markedly with the definition of subitizing which envisages a near perfect performance regardless of the numerical ratio (Kaufman et al., 1949). Indeed, we obtained a pattern similar to the classic definition of subitizing for low numbers only in the sequential presentations (Figure 4). These show a very shallow dependence of performance on numerical ratio, in particular for the IES measure of Figure 4C. Such shallow pattern, however, is not found elsewhere. If the simultaneous presentation simply added noise to the judgment, one would have expected near flat performance also in the results for Condition A and C. However, Figures 2C and 6C instead show a strong dependence on numerical ratio that is incompatible with this view. Consistently, no interaction was observed between simultaneous tasks for small and moderate number ranges (Condition A/C vs. B). The most important characteristic of subitizing disappeared. In addition, lack of interaction suggests that simultaneous comparison between small numbers follows a psycho‐physical law similar to those for moderate numbers, which implies a shard neural mechanism (i.e., numerosity).

Another fact is that the individual IES of simultaneous subitizing and estimation are significantly correlated (Condition A vs. B). In theory, a correlation could occur for several reasons. In particular a high correlation could be the result of observers relying on the same perceptual mechanism to solve two tasks or could be the result of the fact that individual performance is strongly determined by common non‐perceptual factors (such as capacity to allocate attention over space). However, a look at all the five conditions tested may be helpful. On the one hand, if we assume that the IES correlation is mainly due to shared attentional or perceptual processes, then the lowest correlation coefficient is expected between conditions with the most differences. According to Table 1, significant correlation for two number ranges can even be observed between non‐overlapping comparison and overlapping comparison (C vs. B); or between simultaneous subitizing and sequential numerosity (A vs. E; B vs. E), although stimulus presentation differs under several aspects. On the other hand, if we assume that the IES correlation is attributed to individual cognitive capabilities, then the correlation is expected especially between similar conditions. However, no correlation was found between D and E, despite they being identical in all aspects except the number range. Therefore, correlations repeatedly revealed between simultaneous comparison tasks within the subitizing range and comparisons within the numerosity range indicate that the simultaneous enumeration within the subitizing range is based on the estimation mechanism, rather than alternative shared processes.

In this study, it is clear that subitizing is impaired in simultaneous comparisons, in which one has to divide one's attention onto both subsets to compare the item number. No extra working memory load is supposed to be taxed compared to sequential comparisons, because only one binary answer “red” or “green” is proposed to be stored in each trial in simultaneous tasks. Simultaneous comparison may strain both the perceptual and attentional resources. Which factor is dominant in inducing the impairment? A low level degradation of inputs could be caused by the distractors, which are spatially overlapped with the targets in Condition A; however, this perceptual explanation is not sufficient because similar impairment is still revealed in Condition C, in which the perceptual noise is much lower as the targets and distractors are spatially separated.

In the current study, identical stimuli duration was adopted for all conditions (250 ms). It has been suggested that 100–150 ms is necessary for subitizing, and that enumeration for small numbers will be carried out by approximate estimation when duration of stimuli is extremely brief (Melcher et al., 2021). Limited stimuli duration cannot explain the impairment for subitizing (Condition A & C), because errorless subitizing survives in sequential task (Condition D) with the same duration. Rather than a limited duration resulting in incomplete processing in simultaneous tasks, the dissociation between D and A/C should be explained by the lack of parallel processing capabilities in subitizing. As errorless subitizing endures in sequential tasks, on the assumption that subitizing has the capability of parallel processing (Halberda et al., 2006; Liu et al., 2020), then subitizing should also be demonstrated in simultaneous tasks. However, it is disrupted and superseded under these conditions.

On the other hand, attention loading may provide a plausible explanation for the results. Subitizing may be based on attentive mechanisms that can achieve precise enumeration by tracking stimuli (Burr et al., 2010), and dividing attention into two sets can hamper such mechanisms. Hence, subitizing may possess an innate limitation. Namely, it cannot process subsets that are simultaneously presented. In any event, even without knowing the precise type of attention involved, it is clear from the present data that one of the most outstanding feats of the human numerosity system, that of precisely appreciating very small numerosities, is indeed fragile and can be knocked out by the simple presence of distractors.

Note that most studies examine subitizing in an estimation task (but also see the studies of Feigenson & Carey, 2003, and Pomè, Anobile, Cicchini, & Burr, 2019). It is suggested that the comparison task may be mediated by the analysis of continuous magnitude rather than subitizing or estimation. However, ER and IES dissociation was found in sequential comparison tasks between number ranges, as well as between sequential and simultaneous tasks with small numbers (1–4), demonstrating the existence of another mechanism distinct from that of estimation, which is highly related to subitizing. In the control experiment, the circle size was decreased to 56%, so the visual cue of accumulative area was invalid in the comparison. Error rates are around 25% in simultaneous comparison with ratios 0.67 and 0.75. Compared with the formal experiment with an ER around 20% at ratios 0.67 and 0.75, the increase in ER may indicate the influence of visual cue inconsistency. However, the fact that number comparison can be affected by visual cues does not preclude the existence of a specialized numerosity mechanism. Furthermore, the RT and IES in the control experiment are even slower than those in the formal experiment, suggesting that even with conflicting visual cues (e.g., accumulative area and perimeter), the efficiency of number processing remains unimpaired. Importantly, a similar ER, RT, and IES interaction was again found in the control experiment. There is no evidence supporting that the interaction between simultaneous/sequential comparison and small/moderate numbers, which was revealed both in the formal and control experiments, was due to the processing of continuous magnitude rather than discrete numerosity.

There is an increase in reaction time for sequential comparison, compared with that for simultaneous comparison, both in the range of subitizing and estimation. This suggests a processing cost associated with the task. Note that the RT cost is much higher in subitizing (770 vs. 630 ms for the smallest ratio) than in numerosity (670 vs. 590 ms). The cost may reflect the processing of recalling in sequential comparison, by which we can compare the first stimuli with the second one. To explain why the RT increase is much higher in the subitizing range, we hypothesize that there may be an extra stage in which the presentation is transferred from perceptual format to auditory or verbal format, and the comparison may be conducted with the verbal format. In sequential comparison, the non‐symbolic stimuli (e.g., three dots) are represented as “three” when the stimuli showed up. Then, the comparison is based on symbolic rather than non‐symbolic representation. Spontaneous naming, which is the most prominent trait of subitizing, may be responsible for the RT rise in sequential comparison for small numbers. Importantly, the spontaneous naming is not suggested in simultaneous comparison, as the RT for small numbers is equal to that for moderate numbers. This result provides converging evidence supporting that subitizing endures in sequential rather than simultaneous tasks.

This study also reinforces the idea that the estimation system is not limited to sets of moderate numerosity and handles also very low numerosities. This reinforces our previous studies (Liu et al., 2020). Hence any theory that aims at performing estimation has to include the capability of estimating also 1–4 items. It is particularly difficult that at such low numerosities, a mechanism based on “extensive measures,” such as spatial extent and density, could operate. It is also unlikely that estimation for very small numerosities could be done by ink (i.e., by comparing the cumulative area of dots). In the control condition, the circle size was reduced by 44% so that the area cue became invalid in comparison tasks. Still, similar results in ANOVA were revealed. Therefore, the notion of a direct number sense is supported by the current study showing that estimation is acting even with very few items.

## CONFLICT OF INTEREST STATEMENT

The authors declare no conflict of interest.

## ETHICS STATEMENT

The studies involving human participants were reviewed and approved by the Ethics Committee of Yunnan Minzu University. The participants provided their written informed consent to participate in this study.

## Data Availability

The original contributions presented in the study are included in the article/supplementary material. Further inquiries can be directed to the corresponding author.
